# Pose Estimation and Damage Characterization of Turbine Blades during Inspection Cycles and Component-Protective Disassembly Processes

**DOI:** 10.3390/s22145191

**Published:** 2022-07-11

**Authors:** Philipp Middendorf, Richard Blümel, Lennart Hinz, Annika Raatz, Markus Kästner, Eduard Reithmeier

**Affiliations:** 1 Institute of Measurement and Automatic Control, An der Universität 1, 30823 Garbsen, Germany; lennart.hinz@imr.uni-hannover.de (L.H.); markus.kaestner@imr.uni-hannover.de (M.K.); eduard.reithmeier@imr.uni-hannover.de (E.R.); 2 Institute of Assembly Technology, 30823 Garbsen, Germany; raatz@match.uni-hannover.de

**Keywords:** borescopic fringe projection, feature segmentation, pose estimation, film-cooling holes, point cloud registration

## Abstract

Inspection in confined spaces and difficult-to-access machines is a challenging quality assurance task and particularly difficult to quantify and automate. Using the example of aero engine inspection, an approach for the high-precision inspection of movable turbine blades in confined spaces will be demonstrated. To assess the condition and damages of turbine blades, a borescopic inspection approach in which the pose of the turbine blades is estimated on the basis of measured point clouds is presented. By means of a feature extraction approach, film-cooling holes are identified and used to pre-align the measured point clouds to a reference geometry. Based on the segmented features of the measurement and reference geometry a RANSAC-based feature matching is applied, and a multi-stage registration process is performed. Subsequently, an initial damage assessment of the turbine blades is derived, and engine disassembly decisions can be assisted by metric geometry deviations. During engine disassembly, the blade root is exposed to high disassembly forces, which can damage the blade root and is crucial for possible repair. To check for dismantling damage, a fast inspection of the blade root is executed using the borescopic sensor.

## 1. Introduction

**Figure 1:**
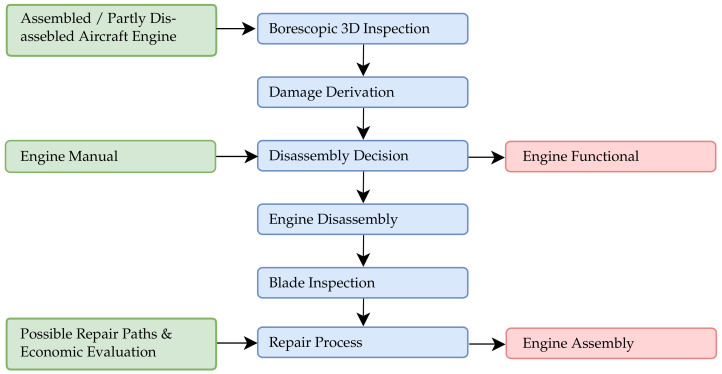
Workflow of the researched maintenance process with focus on the engine inspection and disassembly.

The Collaborative Research Center 871 “Regeneration of Complex Capital Goods” is conducting research into the sustainable operation and resource-efficient repair of capital goods using the example of aero engine components. To this end, research is being performed on the optimization of the process chain of a maintenance interval starting from the early fault detection on the assembled engine. Therefore, the disassembly process, repair options using laser buildup welding and thermal spraying, as well as final contouring and the associated economic feasibility studies and risk assessments are being investigated [1]. This article focuses on the beginning of the process chain, where early defect detection and component-protecting disassembly is researched. In line with the current industry standards, the maintenance cycle of aircraft engines is based on manual visual inspections of the assembled areo engines using borescopes and video endoscopes. In the case of a subjective assumption of damage (on individual components, such as turbine blades), the engine is disassembled, and each individual component is visually inspected. Once the engine is fully disassembled, a 3D measurement of the engine’s components can be carried out using a coordinate measuring machine or industrial laser triangulation or fringe projection sensors. However, these inspections are only possible in the dismantled state and have already resulted in high costs. Within the scope of this paper, a borescopic 3D measurement system was developed for the inspection within assembled and partially disassembled aero engines. Using these measurement systems, the condition of turbine blades can be quantified precisely and possible damages can be derived. Thus, it is possible to measure the characteristic damage shape, size and depth metrically and to compare these with the permissible limits. This provides an objective data basis for necessary dismantling and repair decisions. In addition to the operational wear of the turbine blades, safety-relevant areas, such as the blade root, must also be examined for further repair decisions and selection of the repair process. In the area of the blade root, deformations can occur due to high disassembly process forces, resulting in a loss of firmness of the mechanical connection. For this purpose, an optical measurement of the turbine blade root in the disassembled state is performed. Figure 1 shows a brief overview of the maintenance process investigated, focusing on the inspection of aircraft engine components.

In order to establish a standardized inspection and disassembly decision process for aero engine maintenance, these processes must be as (sensor) data driven and human independent as possible. In this context, standardization of the inspection process is to be achieved by reproducible positioning of endoscopic sensors as well as through an objective and consistent evaluation process by means of high-resolution optical 3D measurements (fringe projection profilometry). In industrial environments, inspection in confined spaces is carried out manually by trained personnel. Using a rigid borescope or a video borescope, a visual inspection is performed, and the component condition is subjectively assessed. This subjective evaluation can be error prone and inconsistent, compared to industrial measurement systems but offers better flexibility in quality assurance [2,3,4]. To limit this challenge, many borescopic 2D inspection approaches have been extended with cameras. This allows defect segmentation and classification based on 2D image data using neural networks and support vector machines. The field of automated defect detection and decision support based on 2D image data has therefore already been researched [5,6,7,8,9,10,11,12,13,14,15]. Due to a strong miniaturization in the field of camera sensors and fiber optics, the first endoscopic 3D measuring devices were able to be developed in recent years. Besides the classical 2D video endoscopes, endoscopic approaches based on stereoscopic 3D imaging, phase shift profilometry, laser light section method and shape from focus have been researched. Geng et al. accordingly published a review of available endoscopic 3D imaging techniques [16]. In the commercial sector, only a few sensors using stereoscopic 3D imaging and phase shift profilometry are available. In the field of medical technology, rigid endoscopic designs are required for use in surgery: for instance, the stereoscopic 3D system (IMAGE1 S™ 3D) from KARL STORZ SE & Co. KG (Tuttlingen, Germany) and ENDOEYE 3D Rigid VIDEOSCOPE from Olympus Medical Systems Corp. (Shinjuku, Japan). Due to their use in surgery and the nature of the application, these measurement systems are designed for viewing angles of 0 to 30 degrees. However, for rigid endoscopic inspection inside an aero engine, a camera viewing direction of approximately 90${}^{{}^{\circ}}$ is required. For the inspection in industrial environments, Baker Huges (Baker Hughes a GE Company, Houston, TX, USA) developed the Mentor Visual IQ, a flexible, actuatable 3D measurement system based on phase shift profilometry. It allows a manual geometric evaluation of small component areas, using a handheld control unit. More complex data evaluation and interfaces for quality assurance based on CAD data are not available with current industrial sensors. In addition, the design of the available sensors is not directly adaptable to the automated inspection within aero engine maintenance intervals. Instead, a borescopic fringe projection sensor design enables precise navigation and high-precision 3D measurements in confined spaces and addresses the special requirements of engine inspection. Schlobohm et al. were able to implement inspections of engine blisks using this sensor design and demonstrate the automation capability [17,18]. In addition to the aforementioned requirements, a metric deviation analysis must be performed in order to assess the condition of engine components. Each defect and its location must therefore be compared to the limits specified in the respective engine manual (V2500 Engine Manual, Borescope Inspection [19]). To perform this assessment, the optical measurement must be registered to a reference geometry in order to quantify common types of damage on turbine blades, such as erosion, cracks, nicks, dents, burns, leading edge burn through, trailing edge metal loss and coating damages. Due to the rotational degree of freedom of the engine shaft, the relative position of each turbine blade to the measurement system is unknown. This is critical for the evaluation of the measurement data, since simple alignment approaches using iterative closest Point (ICP) algorithms are not robust. Therefore, one of the methods presented in this paper is a robust pose estimation method for turbine blades based on 3D measurements.

**Figure 2:**
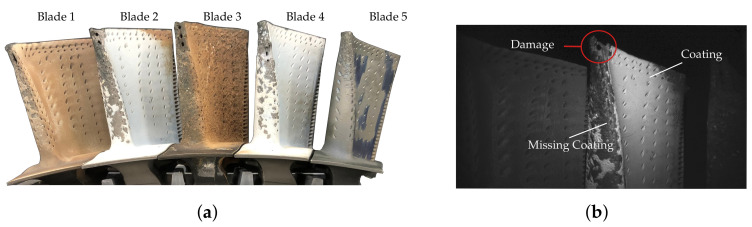
Turbine blades with different levels of wear and damages and a camera view of an exemplary inspection pose. (**a**) Blade setup for borescopic measurements. (**b**) Camera view of an exemplary measurement pose.

Figure 2a demonstrates the arrangement of turbine blades with different exemplary defects used for the investigations. Blades 1 to 4 show heavy use with defects at the leading edge. In addition, blades 1, 2 and 4 have missing coating on the leading edge, and the entire coating of blade 5 is burnt. Burning of the thermal coating results in a very rough, porous surface. In contrast, blade 5 has already been repaired and is decoated. The following segmentations and evaluations refer to measurement poses similar to Figure 2b, which demonstrates a typical image captured with a borescopic sensor. Correspondingly, turbine blade 2 is in the center of the measuring volume, and parts of blades 1 and 3 can be seen. The non-uniform and poor illumination as well as the low imaging quality of the miniaturized cameras lead to a relatively low contrast within the image data, which is a common problem during endoscopic inspections [20].

This article is structured as follows. First, the design and principle of the borescopic sensor are explained. In Section 3, a proposal for robust measurement data registration and pose estimation is shown, using the geometric properties of film-cooling holes of turbine blades. For this purpose, a feature extraction approach for film-cooling holes is presented, which allows to perform a pre-alignment of the measurement data. Subsequently, a fine adjustment of the measurement data is carried out, and a damage derivation in the form of geometric deviations is determined. Finally, in Section 4, the inspection of potential disassembly damages is investigated, and an approach for the component-preserving disassembly of engine parts is evaluated.

## 2. Borescopic Fringe Projection

**Figure 3:**
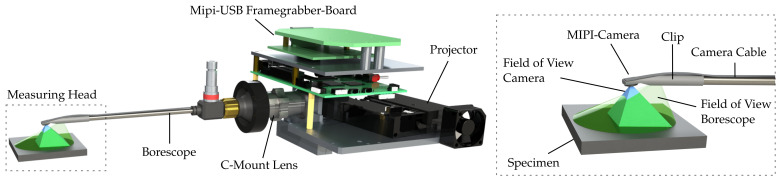
Functional schematic and essential components of a borescopic fringe projection system and a close-up of the measuring head.

For fast and high-precision inspection in confined spaces, the measuring method of fringe projection profilometry is adopted to a borescopic sensor design. Schlobohm et al. introduced this sensor design, which enables the spatial separation of measuring head and projection and evaluation devices while keeping a rigid sensor setup [17]. This, in turn, enables a precise navigation into maintenance openings and cross sections of less than 9 mm diameter, which then allows for automated inspections in difficult-to-access areas. The functional schematic and a basic setup of a borescopic fringe projection sensor is illustrated in Figure 3.

To ensure better readability in the following, a detailed product description and their manufacturers are listed in Table 1. The optical connection to the measuring head is achieved by using borescopes within the projection path as well as miniaturized Chip-on-the-Tip cameras. Sinusoidal patterns are sharply imaged on the specimen surface by projecting fringe patterns through a borescope by means of a C-Mount lens. This allows the use of high-resolution micromirror device projectors that can be adapted and triggered for the use in the field of fringe projection, as published in [21]. In order to implement sensor heads for different applications, borescopes based on the Hopkins rod-lens system with various diameters of 4 mm to 6.5 mm and lengths of 110 mm to 300 mm are used as the optical connection between the measuring head and the projector [22]. The measurements and results shown below were obtained using a borescope with a diameter of 6.5 mm, a length of 300 mm, a view direction of 70${}^{{}^{\circ}}$ and an aperture angle of 80${}^{{}^{\circ}}$. Compared to the smaller diameter borescopes (e.g., 4 mm), these are stiffer and allow for increased light input, thus providing better illumination. The total diameter of the measuring head is dependent on the size of the borescope, as the Chip-on-the-Tip camera is attached to the borescope using a 3D printed clip. Based on the clip design and the alignment along the borescope axis, the triangulation base between camera and projector can be flexibly adjusted and therefore allows a flexible adaptation of the working distance of the sensor. A working distance of 4–6 cm is used for engine inspection, which also represents the distance from the maintenance opening to the turbine blades. The use of a stereolithographic 3D printer allows to print wall thicknesses below 1 mm, resulting in a sensor head diameter of 8 mm. In other sensor configurations, measuring head diameters of 6.5 mm could also be implemented; however, the size of the camera module is a limiting factor in this setup. In order to project sinusoidal fringe pattern, a modified DLP 4500 EVM micromirror device is used. The digital micromirror device (DMD) forms the pattern by binarily tilting individual micro mirrors at a resolution of 912 × 1140 px. A modification of the projector enables to install a C-Mount lens (f=38 mm) to the projection unit and thus to project the fringe patterns into the borescope. To record the projected patterns, a miniaturized 2-megapixel multimedia sensor OV2740 is applied. The Chip-on-the-Tip design with the serial cable connection of the camera and lens (f=1.83 mm/F8.0) were manufactured by MISUMI Electronics Corp. (New Taipei, Taiwan). The serial connection of the Mobile Industry Processor Interface (MIPI)-camera (without evaluation electronics on the chip) enables very small dimensions, allowing the two megapixel sensor to have a volume of 5 mm × 5 mm × 3 mm. A MIPI-USB frame grabber board based on the EZ-USB CX3 USB 3.0 peripheral controller from Cypress Semiconductors Corporation (San Jose, CA, USA) is used to control the miniaturized camera sensor. The camera is supplied with the appropriate clocks, voltage levels and data lines by means of a specially developed daughter board. Using this frame grabber setup, an uncompressed raw signal (RAW10) at 60 fps can be obtained from the sensor. Other types of sensor packaging often provide compressed video signals (e.g., mp4), which contain compression artifacts and further loss of image quality [23].

Due to the highly miniaturized optical components, the captured images are highly affected by noise, nonhomogeneous illumination and strong distortions [22]. To counteract the illumination issue, a high dynamic range (HDR) approach is used during the multi-frequency phase shift measurements. The camera is calibrated according to the pinhole approach of Zhang et al. using a low reflection dot grid target [24]. Subsequently, the projector is calibrated as an inverse camera and the radial and tangential distortion of camera and projector are calculated using the polynomial approach of Conrady and Brown [25]. Finally, a stereo calibration of camera and projector is performed. In order to estimate the reconstruction quality of the borescopic sensors, the probing error with respect to size and form was determined for endoscopic sensor assemblies [26]. According to VDI/VDE 2634-2 [27], a cylindrical feature of a calibrated microcontour standard was measured at a working distance of 30 mm, resulting in a probing error of 20 μm. The probing error with respect to size on this feature was determined to be 40 μm using 20 repeated measurements, following the guideline JCGM 100:2008 [28]. The radius of the calibrated microcontour standard is 5994.0 μm according to the calibration certificate. Please refer to [26] for further accuracy and measurement uncertainty investigations. Additionally, when measuring surfaces with limited optical cooperativity, the known physical limits of triangulating optical measurement principles apply.

## 3. In-Situ Inspection of Turbine Blades

**Figure 4:**
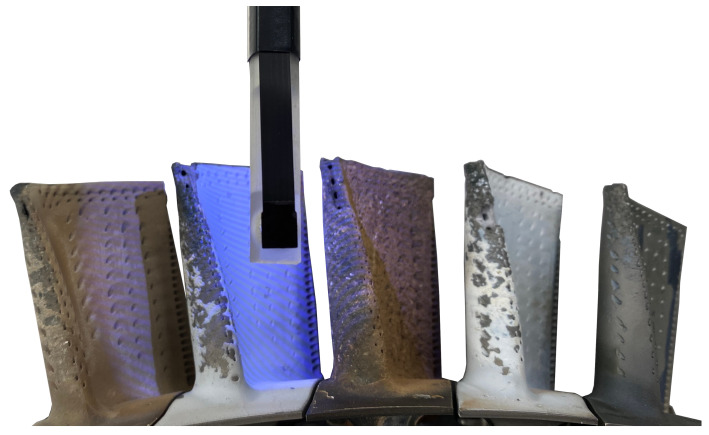
Borescopic Inspection of turbine blades using a 8 mm sensor head.

The borescopic measuring head is shown in Figure 4; it demonstrates a fringe projection measurement of the academic test setup for engine inspection. Here, the borescopic measuring head is positioned approx. 4 cm above the turbine blades and projects fringe patterns onto its surface. Next, the leading edge in particular is examined, as the most damage occurs at this location [19]. This setup represents the approximate measurement pose within the aero engine, whereby the measurement system can also be rotated to capture different areas of the blades.

Further, the robust condition assessment of turbine blades based on 3D measurement data requires a reliable pose estimation of individual turbine blades (in the measurement data). Due to the unknown position and orientation of the turbine blades to the sensor, simple point cloud registration approaches (using ICP) often converge to local minima. Consequently, incorrect measurement data alignment is the basis for deviation analysis, resulting in faulty condition assessments. In order to estimate the specimen’s pose even after operational stress, a feature detection approach for the identification of film-cooling holes of turbine blades is presented. These film-cooling holes are unique features and have a defined distance between each other, as well as a characteristic geometric shape. To perform a pose estimation, first the film-cooling holes of a reference geometry (CAD geometry or reference measurement) are detected, and coordinates of these features are assumed as a reference pattern. By means of an equivalent feature detection within the borescopic measurements, film-cooling holes are then identified. Using the obtained feature sets, a pose estimation is performed, which is used to pre-align and mask the reconstructed point clouds of the borescopic sensor. Using the roughly aligned point clouds, a robust registration approach can be implemented as follows. Accordingly, a novel film-cooling hole detection approach is presented, followed by a multi-stage point cloud registration process.

### 3.1. Film-Cooling Hole Detection

**Figure 5:**
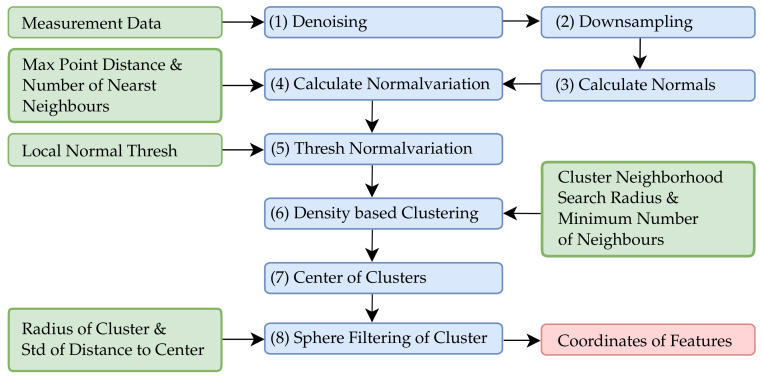
Workflow of the film-cooling hole identification.

An overview of the algorithm sequence used for the film-cooling hole extraction is shown in Figure 5. Green boxes indicate input data and manual inputs for algorithms, blue boxes represent the implemented functions and red boxes imply obtained results. To ensure a simple text-image correlation, the implemented and applied algorithms from Figure 5 and following are labeled with an ascending number. Based on the reconstructed point clouds, a denoising (1) and uniform down-sampling (2) is performed to obtain comparable initial data. This allows a sensor specific but consistent parameterization in subsequent algorithms. During point cloud denoising, the average point distance in a neighborhood of four is first determined and the standard deviation of the point distances is calculated. Points with a distance above the one sigma interval are then removed as outliers. The downsampling of point clouds is performed using a box grid filter approach, where the entire point cloud is divided into grid boxes of a manually defined size. Within a box, all points are merged into a single one by averaging the coordinates and corresponding normal vectors. In this way, the shape of the actual point cloud is preserved as much as possible.

To separate the geometric properties of film-cooling holes from nominal contours and curvatures of the turbine blade in the point clouds, the variation of normal vectors in a local neighborhood is analyzed. For this purpose, the normal $n_{i}\in R^{3}$ of each point $p_{i}\in R^{3}$ of the point cloud $\mathrm{pc}\in R^{3\times N}$ is estimated first [29], by which a global sensor center $p_{s}\in R^{3}$ is introduced due to the ambiguous surface orientation after local plane fitting (3). Additionally, the case distinction

(1) $$n_{c,i}=\left\{\begin{array}{cc} n_{i}, & \mathrm{if}\alpha_{i}>\frac{\pi }{2}\;\mathrm{or}\;\alpha_{i}<-\frac{\pi }{2}.\\ -n_{i}, & \mathrm{otherwise}. \end{array}\right.$$

is made for each surface normal $n_{i}$, where

(2) $$\alpha_{i}=atan2\left(\begin{array}{c} ∥\left(p_{i}\times p_{s}\right){∥}_{2}\;,\\ p_{i}\;\cdot \;p_{s} \end{array}\right)$$

describes the spatial angle between $p_{s}$ and each object point $p_{i}$. This is based on a *rangesearch*-classification: $R^{3\times N}\times R^{3\times N}\to N^{N\times k_{i}}$, which identifies the indices $\iota_{i}\in N^{k_{i}}$ of the subset of points ${\mathrm{pc}}_{i}\subset \mathrm{pc},\left|{\mathrm{pc}}_{i}\right|=k_{i}$ for each query point $p_{i}$ with

(3) $$\iota_{i}=\{j\;|\;∥p_{i}-p_{j}{∥}_{2}<d_{c}\;\forall \;\underset{i\neq j}{p_{j}}\in \mathrm{pc},\;j=1,\ldots ,N\}$$

within a sphere for cut-off distance $d_{c}$ = 0.25 mm with $\iota_{i}=\left\{\iota_{i,1},\ldots ,\iota_{i,k_{i}}\right\}$. For performance requirements, the *rangesearch* is performed using a basic K-NN search (here: *K* = 100) based on the fast *k*-d search tree [30] with subsequent masking. The metric for quantifying the local surface normal variation

(4) $$\zeta_{i}={∥\left\{w_{n,i,1}\;\cdot \;\left(n_{c,i}-n_{c}\left(\iota_{i,1}\right)\right),\ldots ,w_{n,i,k_{i}}\;\cdot \;\left(n_{c,i}-n_{c}\left(\iota_{i,k_{i}}\right)\right)\right\}∥}_{2}$$

is obtained from the normalized weighted vector norm of all local normal variations (4), where the weights

(5) $$w_{i,j}=e^{\frac{-d_{ij}^{2}}{d_{c}^{2}}}$$

are determined via a Gaussian membership function with respect to cut off distance $d_{c}$ and point distance

(6) $$d_{ij}={∥p_{i}-\mathrm{pc}\left(\iota_{i,j}\right)∥}_{2}$$

with $j=1,\ldots ,k_{i}$. To account for the different number of neighboring points $k_{i}$, with respect to each query point $p_{i}$, a final normalization is performed:

(7) $$w_{n,i,j}=\frac{w_{i,j}}{\sum_{p=1}^{k_{i}}w_{i,p}}.$$

**Figure 6:**
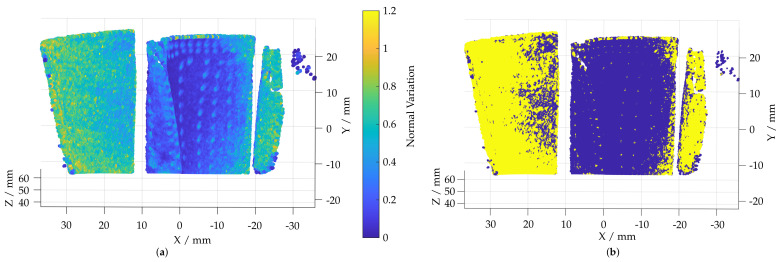
Visualization of the normal variation approach used to identify film-cooling holes. (**a**) Normal variation ζ plotted on the reconstructed point cloud. (**b**) Threshold applied to the calculated normal variation. Areas with strong changes in normal direction colored yellow.

Figure 6a shows the resulting local normal variation. By means of a threshold value, the areas of strong geometric changes are selected (5); see Figure 6b.

**Figure 7:**
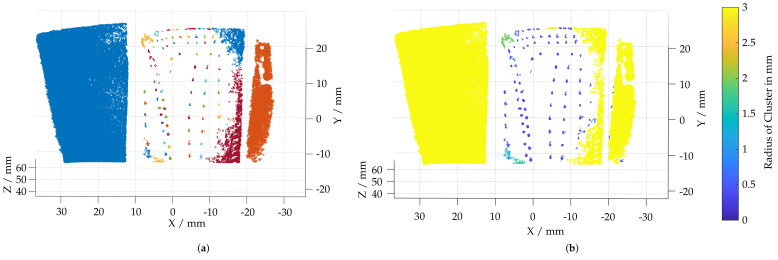
Visualization of the clustering approach used to separate film-cooling holes. (**a**) Clusters calculated using a DB Scan algorithm. (**b**) Radius of an evolving sphere fitted to each cluster.

In order to separate the selected areas and assign them to the individual film-cooling holes, a density-based clustering procedure DB Scan [31] is carried out (6). By limiting the neighborhood with a search radius and a minimum number of points required for a cluster, the threshed areas with strong changes in normal directions are divided into clusters in Figure 7a. In addition to the classification into individual clusters, the geometric shapes of the film-cooling holes must be considered to remove outlier clusters. For this, the center of the cluster is calculated by the mean location of all points within the cluster (7). A spherical enveloping geometry can be used to account for the real geometric shapes of the film-cooling holes. These have a defined maximum size, a defined distance between them and a characteristic shape. For this purpose, a spherical fit is performed at the center of each cluster and the radius of the cluster is determined (8). Figure 7b plots the resulting cluster radius. To consider the shape of the cluster, the standard deviation of the distance of each point to the cluster center is used as an additional filter criterion.

**Figure 8:**
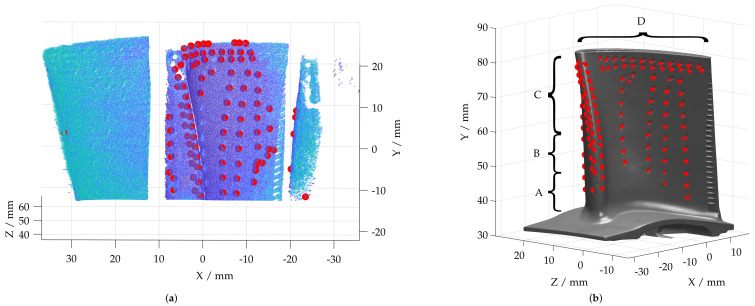
Detected film-cooling holes within borescopic measurement and reference geometry. (**a**) Film-cooling holes detected in the borescopic measurement. (**b**) Film-cooling holes detected in a reference measurement with a mapping of the inspection areas A–D from the engine manual.

After filtering, few clusters meet the geometric conditions of the film-cooling holes. In the area of the damaged leading edge and the tip of the blade, additional faulty features are detected; see Figure 8a. In comparison, the feature detection applied on a reference measurement of a disassembled, undamaged blade with an industrial sensor is shown in Figure 8b. The identified film-cooling holes derived from the reference measurement will be used as a reference pattern for the subsequent pose estimation.

In addition to the segmented film-cooling holes, the turbine blade in Figure 8b is divided into sections A–D. According to the engine manual and thus as a basis for borescopy, these areas are used to define the location of damage within the blade. Area A comprises the lower 25% of the blade without a leading and trailing edge. Since the highest mechanical load is applied here and it represents the connection between the blade root and the blade, the permissible damage and form deviations are smallest in this area. As the distance from the blade root increases, the permissible damage size increases. Area B includes the area from 25% to 50% of the blade’s height as well as the area of the trailing and leading edge from 0% to 50% of the blade. The remaining part of the blade is referred to as area C and the blade tip as area D. However, it is not possible to measure area D in the assembled state, as it is outside the field of view of the measuring system.

For extremely worn blades, this feature detection approach may not be appropriate because the coating is burned or flaked off. It can therefore lead to frequent changes in the normal vectors, which results in a normal variation with many outliers and limits the segmentation of film-cooling holes. However, with such strong variations in the normal vectors, it can be assumed that the turbine blade is damaged.

### 3.2. Data Registration

**Figure 9:**
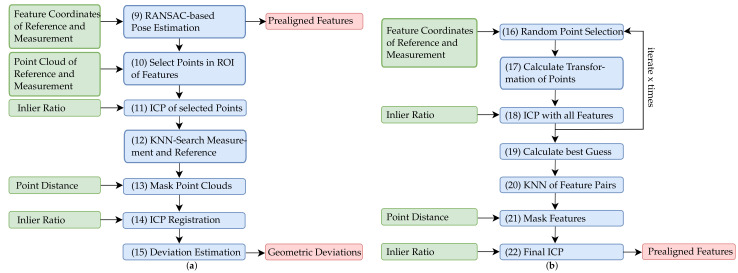
Workflow of the data registration after film-cooling hole segmentation. (**a**) Overview of the data registration approach. (**b**) Sequence of the RANSAC-based pose estimation.

The two feature sets obtained (endoscopic measurement and reference geometry) are used to numerically determine the pose of the turbine blade. Since not all film-cooling holes can be detected and some also may be faulty, a best-fit of these feature sets has to be performed. A random sample consensus (RANSAC [32])-based approach is applied to fit the features of the endoscopic measurement into the reference feature set. Figure 9a gives an overview of the data registration algorithm, while Figure 9b depicts the RANSAC-based transformation estimation in detail (9).

The transformation estimation is a two-step process. In a first step, an initial transformation is computed based on three randomly selected features of the reference feature set $r\in R^{3\times N}$ and the measurement feature set $m\in R^{3\times N}$ (16). The transformation is calculated using an implementation according to Hinz et al. [33] (17). The algorithms are based on the work of Besl et al. [34] and Kabsch et al. [35]. Therefore, first the centroid of the three selected features per feature set is calculated using Equation (8):

(8) $$\mu_{m}=\frac{1}{n_{m}}\sum_{i=1}^{n_{m}}m_{i}\;and\;\mu_{r}=\frac{1}{n_{r}}\sum_{i=1}^{n_{r}}r_{i}.$$

According to [34], the square cross-covariance matrix $\sum_{mr}$ of Equation (9) can be used to find the optimal rotation between the feature sets using the singular value decomposition, $\sum_{mr}=U\sum V^{T}$:

(9) $$\sum_{mr}=\frac{1}{n_{m}}\sum_{i=1}^{n_{m}}\left[\left(m_{i}-\mu_{m}\right){\left(r_{i}-\mu_{r}\right)}^{T}\right]=\frac{1}{n_{m}}\sum_{i=1}^{n_{m}}\left[m_{i}r_{i}^{T}\right]-\mu_{m}\mu_{r}^{T}.$$

Equation (10) describes the resulting rigid body transformation $T_{initial}$ of the selected features:

(10) $$T_{initial}=\left[\begin{array}{cc} R & t\\ \overset{︷}{VU^{T}} & \overset{︷}{\mu_{r}-VU^{T}\mu_{m}}\\ 0^{T} & 1 \end{array}\right].$$

**Figure 10:**
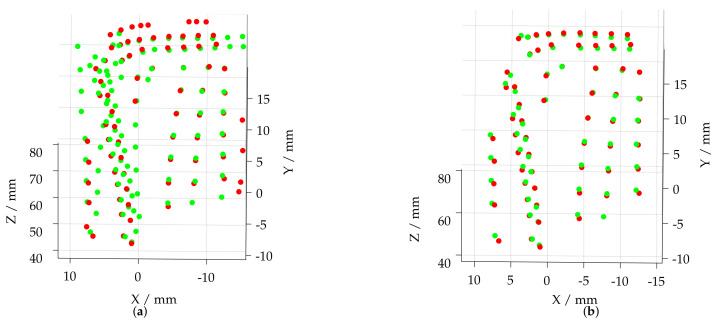
Registration of segmented film-cooling holes of measurement data (red) and reference model (green). (**a**) Features matched after (19). (**b**) Features matched after (22).

After the initial transformation, a subsequent ICP of all features of both feature sets is performed (18). Due to the unknown numbers of outliers, a low inlier ratio (0.5) is applied. Additionally, the root mean squared error of the Euclidean distance between the inlier points of the aligned feature sets is calculated. In order to generate independent starting values (feature coordinates) for the icp optimization and to avoid the permanent convergence of the optimization into local minima, this procedure is frequently repeated (x=50,000 times). The best guess is selected calculating the transformation with the minimum Euclidean distance of the fitted feature sets (19), which is given in Figure 10a. Based on this alignment, the point distance between the feature sets is calculated using K-NN search (K=1) (20). An additional distance threshold of 1 mm is used to filter any unmatched outliers, thus giving $r_{inlier}$ and $m_{inlier}$ (21). This ensures a precise registration of both feature sets by means of an ICP (22), while the feature alignment after registration is given in Figure 10b.

For the final adjustment of the reconstructed point cloud to the reference, two more ICP iterations are performed, (11) and (14). Therefore the reference geometry is re-sampled into a point cloud $pc_{r}$. First, each point $p_{i}\in R^{3}$ of both point clouds (measurement $pc_{m}$ and reference $pc_{r}$) within a distance d = 0.5 mm to the identified inlier features (actual film-cooling holes) are selected by means of a range search (10). Using the selected points

(11) $$N_{m}=\left\{p_{i}\;|\;∥p_{i}-p_{j}{∥}_{2}<d\;\forall \;p_{i}\in pc_{m},\;p_{j}\in pc_{r}\right\}\;$$

and

(12) $$N_{r}=\left\{p_{i}\;|\;∥p_{i}-p_{j}{∥}_{2}<d\;\forall \;p_{i}\in pc_{r},\;p_{j}\in N_{m}\right\}\;$$

an ICP is applied to ensure appropriate consideration of the points in the area of the film-cooling holes (11). For the second ICP iteration, a range search is performed, which is based on all points of the point cloud $pc_{r}$ and the transformed $pc_{m}$ (12). Using a distance threshold of 0.5 mm, the point cloud $pc_{m}$ is then masked (13). Finally, all inlier points of $pc_{m}$ are registered to the reference point cloud $pc_{r}$ using an ICP with a strict inlier ratio (0.9) (14).

### 3.3. Damage Derivation

**Figure 11:**
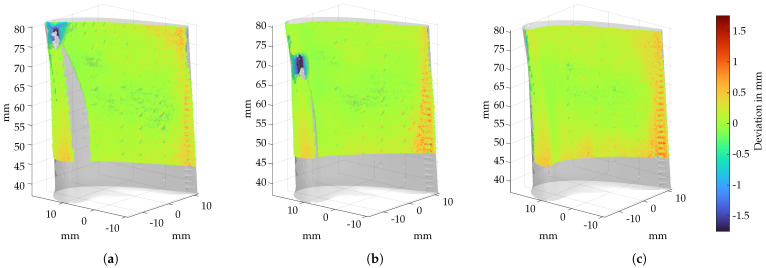
Deviation analysis using the example of 3 turbine blades, where the point deviation is calculated to a reference geometry of an healthy blade. (**a**) Point deviation of turbine blade 2. (**b**) Point deviation of turbine blade 4. (**c**) Point deviation of turbine blade 5.

After registration, the deviation of the borescopic measurement to the reference geometry is determined in the polygonal normal direction (15). To assign the specific (polygon) planes to the corresponding 3D points of the measurement, a reference point cloud is generated from all polygons. A K-NN (K=1) classification of the reference point cloud and the reconstructed point cloud are carried out, and the point deviation of all points is determined to the nearest polygon. Figure 11 shows the geometric deviations of three exemplary measurements of turbine blades 2, 4 and 5. Figure 11a,b visualizes the missing material at the leading edge within area *C* of the blade. In contrast, the regenerated blade in Figure 11c shows no damage, meaning that the geometry deviations are within the permissible limits.

**Figure 12:**
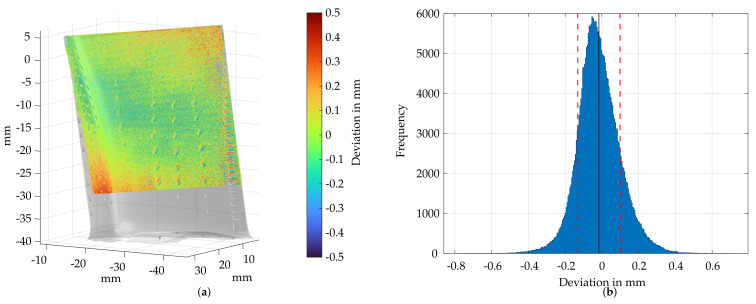
Deviation analysis of the endoscopic measurement compared to a reference measurement of turbine blade 5 using a GOM ATOS Core 200. (**a**) Point deviation of the endoscopic measurement compared to a reference measurement of turbine blade 5. (**b**) Histogram of the point deviation of (**a**).

In the following, a direct comparison of the endoscopic sensor and an industrial fringe projection system was carried out to provide a simplified uncertainty analysis for the combined measurement and registration technique. Using a ATOS Core 200 from Carl Zeiss GOM Metrology GmbH (Braunschweig, Germany), turbine blade 5 from Figure 11c was measured in a disassembled condition with 20 individual measurements, and a reference geometry was derived based on this. Figure 12a shows uses this as a reference geometry on which the endoscopic fringe projection measurement was aligned using the previously presented film cooling hole detection and registration approach. It is noticeable that the geometry deviations especially occur in area of film cooling holes, at the trailing edge and partly at the leading edge. The deviations in the area of film cooling holes are probably due to the artificial closing of film-cooling holes during data processing in the industrial software. In addition, minor registration deviations may be present, which leads to a non-ideal overlap of the measurement and reference geometry in the area of the trailing edge. This causes high deviation values at the flanks of the trailing edge. Figure 12b shows a histogram for the resulting reconstruction deviations. The mean point deviation is 16 μm and the standard deviation is 115 μm. On the basis of these geometry measurements, the subjective decision of the inspectors can be improved by a precise metric evaluation. In the case of damaged blades, an engine disassembly and repair is considered depending on the location, type and size of damage. The use of a borescopic 3D measuring system thus enables a precise assignment of metric geometry deviation and the damage locations on the blade. However, the characterization of the damage type still needs a subjective evaluation, since the classification of the damage types based on point clouds has not yet been performed. In addition, these measurement data can be used to subjectively estimate whether the damaged turbine blade can be repaired. Especially with large damages at the leading edge, a repair is extremely difficult to carry out. In the case of defective turbine blades, a full engine disassembly must be carried out, as the mechanical arrangement on the blade ring requires the disassembly of each individual blade. Accordingly, in the next section, a brief overview of the procedure of component-friendly disassembly and possibilities for the optical inspection of disassembly damages are given.

## 4. Disassembly of Turbine Blades

In order to retrieve the turbine blades to initiate the regeneration process, their repair, replacement or renewal of the blade’s thermal insulation layer, they first need to be disassembled. In general, the disassembly is characterized by uncertainties. Due to a product’s use, e.g., the aircraft engine’s operation, the knowledge about joint tolerances, needed process forces and tool dimensions are lost. However, with varying operational history, the assembly joint’s condition varies widely. Different degrees of solidification mechanisms in the joint, such as fretting or corrosion, are the result of the long service life under tough operating conditions. Depending on the extent of the solidification, a defined force must be applied to the disassembly object in order to set it in motion. Although machines and mechanisms exist to demount the blades, the disassembly is usually executed manually by experienced workers to ensure a component-protecting and -preserving separation of the joining partners. That allows the adaptive adjustment to the operational variation of the joints’ condition.

### 4.1. Component-Protective Disassembly

**Figure 13:**
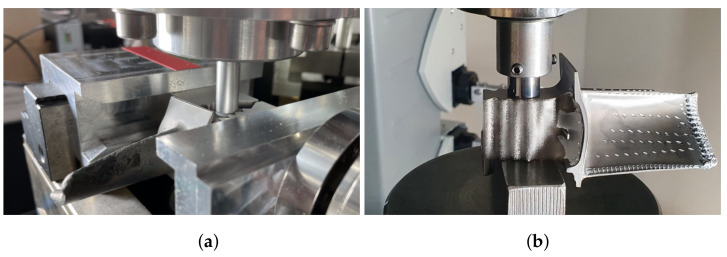
Transfer of the disassembly test setup to the analysis of the maximum load. (**a**) Design of the disassembly test rig: the ram exerts the disassembly force on the blade root. (**b**) Experimental setup of the load tests.

Due to the solidification of the joint, a force (solidification force) is induced, which opposes the disassembly force when the blade root is pushed or pulled out. The disassembly force must not exceed a limit to prevent damage to the blade root but surpass a force minimum corresponding to the solidification to disassemble the blade. That results in a process window within the disassembly force ranges. In order to investigate and execute a component-protective disassembly, a disassembly test rig was developed [36]. Thus, it is possible to record force distributions and describe them as a function of input variables, such as disassembly time, degree of solidification or geometric shape of the blade root. The disassembly test rig consists of a rod which pushes on the blade root with a predefined feed rate in order to move it out of the turbine disk (Figure 13a).

In addition, superimposed vibration on the disassembly movement, induced by a piezo-stack-actuator, is used to reduce the force required to detach the joint. After selecting suitable and optimized setting parameters, it prevents the maximum force limit from being exceeded. Damaging the blade root can thereby be prevented, as outlined subsequently. Regardless of whether the disassembly is executed manually or automatically, exceeding a maximum force must be avoided. Otherwise it leads into damaging the blade root, making the blade unusable for regeneration or reuse. As a result, the blade must be replaced by a spare part, although it can be regenerated or reused without major effort, if it is disassembled gently. Therefore, the load and force limits must be known or determined for each blade model. Due to reasons of confidentiality, no CAD data of the HPT blades were available for the investigation, so that numerical simulations could not be performed. Only 3D reconstructions of industrial sensors are available, but these do not contain any precise information of the internal geometry of turbine blades and the associated wall thicknesses. Therefore, the determination of disassembly damages and component protection was performed experimentally. In the next subsection, the experimental determination of the force limits is described exemplarily.

### 4.2. Determination of the Force Limit

The high pressure turbine blades examined in this work are made of the second generation nickel-base single crystal (SX) superalloy CSMX-4 (CSMX-4 is a registered trademark of the Cannon-Muskegon Corporation, Muskegon, MI, USA) (Ni-6.5Cr-9.0Co-0.6Mo-6.5Ta-5.6Al-6.0W-3.0Re, wt%) [37,38]. It has been extensively studied and documented in the literature and is used in numerous aerospace and industrial gas turbine applications. The application as high pressure turbine blade has demonstrated an impressive combination of high temperature strength, good phase stability and oxidation, hot corrosion and coating performance in extensive engine service [39]. In order to determine the maximum force that can be exerted on the blade root, a load test is performed until plastic deformation is recognized by visual examination. It is necessary to consider that the blade is hollow inside. Figure 13b illustrates the experimental setup. A tungsten carbide ram with a diameter of 12 mm exerts a defined force on the blade root by pushing it linearly into the material, analogous to the disassembly test rig. The applied load is steadily increased up to the target force. When the target force is reached, the ram returns to its initial position, releasing the load. For the examination, several blades that were already disassembled and classified as scrapped parts were used. A step wise increase in the force is executed until plastic material deformation in the form of an imprint of the ram can be detected. The blades used are randomly labeled; the corresponding force stages can be found in Table 2.

**Figure 14:**
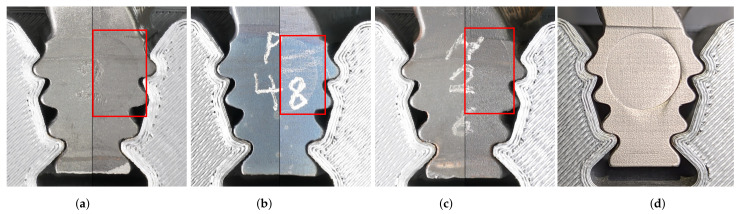
Visual changes due to load exertion on the blade root. (**a**) Disassembly force 50 kN. (**b**) Disassembly force 60 kN. (**c**) Disassembly force 70 kN. (**d**) Disassembly force 100 kN.

**Figure A1:**
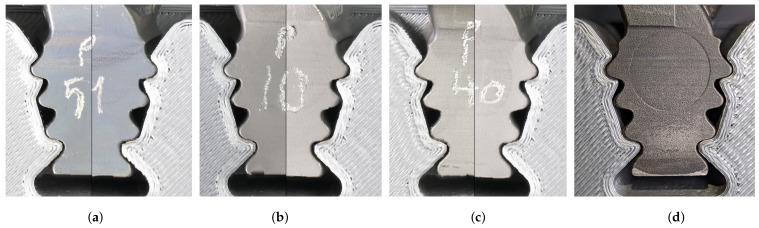
Additional load test with partly visual changes due to load exertion on the blade root. (**a**) Disassembly force 20 kN. (**b**) Disassembly force 30 kN. (**c**) Disassembly force 40 kN. (**d**) Disassembly force 80 kN.

Figure 14 shows, selectively, the results of the load tests using 50, 60, 70 and 100 kN, additional load tests of 20, 30, 40 and 80 kN are given in Figure A1. The left half of the pictures (a–c) show the blade root without any load exertion, and the right half show each blade root after applying the force stages, according to Table 2. Up to 20 kN, hints of traces of the tungsten carbide ram are unrecognizable. From 30 kN on, the imprints become more visible, until the imprint becomes clearly visible above an applied force of 60 kN, as exemplarily illustrated in Figure 14a,b. At a force of 70 kN, a distinct indentation is formed, which shows a palpable plastic material deformation (see Figure 14c, top right corner). By further increasing the force, a clearly visible deformation occurs (see Figure 14d).

### 4.3. Inspection of Blade Roots after Disassembly

In order to perform a fast inspection for disassembly damages, a borescopic fringe projection measurement of the blade root has to be carried out. This is necessary according to regulations: each blade root must be inspected after disassembly and an accept or reject examination must accordingly be carried out. Due to the small blade root and the small depth difference of the plastic deformation, most industrial fringe projection sensors are not particularly suitable for this application. Many of these have a larger measuring volume, resulting in a low axial and lateral resolution related to this application [40]. Due to the smaller measuring volume, the borescopic sensor is applied here; however, other fringe projection sensors can also be used, considering the limitations with regard to resolution and measuring volume. For the borescopic blade root inspection, the turbine blade is positioned in a guidance track. This enables a fixed and repeatable clamping of the turbine blade and ensures that potential deformations are in the field of view. To investigate disassembly damages at the blade root, it is not necessary to register the measurement on a reference geometry and calculate the geometric deviation. In simplified terms, the surface of the blade root can be assumed to be a plane. Any plastic deformation appears as a deviation to this plane.

To perform the deviation analysis, a plane was first fitted into the undamaged surface areas of the blade root of the denoised point cloud. Using the inlier-based plane fit approach according to Smith [41], a reference plane for the blade root is defined by the manual selection of six points. Subsequently, the distances of all points to the plane were calculated, although, theoretically, only geometric deviations in the direction of indentation need to be considered. Due to the blades curvatures at the upper area of the blade root, deviations in opposite direction of indentation can be neglected in this case.

**Figure 15:**
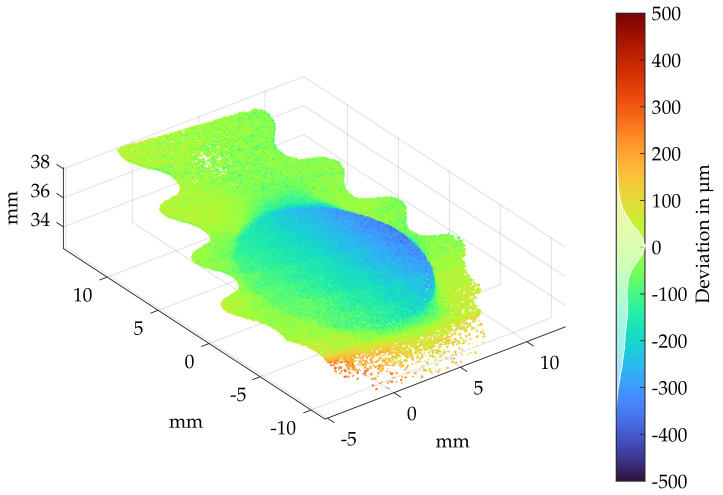
Deviation analysis of the blade root after disassembly forces of 100 kN, Figure 14d.

**Figure 16:**
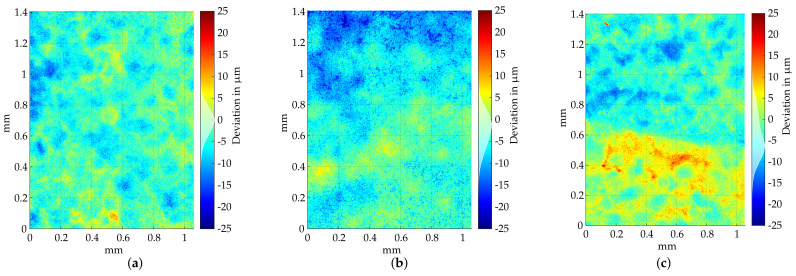
Examination of the surface using a confocal laser scanning microscope. (**a**) Disassembly force 50 kN. (**b**) Disassembly force 60 kN. (**c**) Disassembly force 70 kN.

Figure 15 shows the deviation analysis for significant plastic deformation at disassembly forces of 100 kN. With plastic deformations up to 350 μm, this blade can no longer be repaired and is therefore categorized as scrap. When using lower disassembly forces (<80 kN), very little plastic deformation occurs. As shown in Figure 14, these can still be guessed visually with suitable illumination, but the indentation depth of the stamp is partly below the axial resolution of the sensor and within the sensor noise. For a simple damage check on the blade root, this measuring method is sufficient; however, when examining deformations in the μm range, the use of fringe projection systems is no longer suitable. In order to check at what disassembly force plastic deformation starts, optical measurements were carried out using a confocal laser scanning microscope (VKX 200 from KEYENCE CORPORATION (Osaka, Japan)). Using this measuring method, the investigated measuring volume is significantly smaller, but the axial resolution is below the μm range, enabling the transitions of the stamp impressions to be investigated. Due to the high magnification of the microscope objective (20×), measuring the entire indentation site with a single scan is not possible. Therefore, the transitions between the area of the stamp contact and contactless surface were inspected and described in the following. The calculation of the inlier-based plane fit and the corresponding deviation calculation are performed in a similar method to the fringe projection measurement. During the analysis of process forces below 60 kN (20, 30, 40, 50 kN), the determined deviations indicate no deviations; see Figure 16a. Considering the histogram of the deviation distribution (within the color-bar of Figure 16a), a normal distribution with a standard deviation of less than 5 μm can be determined. Thus, it was not possible to prove the dismantling influences with forces below 50 kN. When increasing the disassembly forces to 60 kN, a slight point to plane deviation in the upper area of Figure 16b can be seen. The histogram plotted onto the color-bar of Figure 16c indicates a divergence of the deviation distribution from the normal distribution. A majority of the points deviate in the direction of the stamp indentation. However, these deviations are very small, meaning a reliable statement regarding plastic deformation cannot be made. Above process forces of 70 kN, stamp marks with an indentation depth of up to 20 μm could be determined; see Figure 16c. The histogram within the color-bar of Figure 16c also reveals a clear deviation from an almost normally distributed undamaged blade root surface. Thus, a plastic deformation of the blade root could be determined from an indentation force of 70 kN, whereby 50 kN should not be exceeded during standard disassembly. For a more specific statement, however, more test series should be carried out, which could not be done due to the limited number of blades.

## 5. Discussion

In this section, the presented borescopic sensor, developed approaches and algorithms for point cloud registration as well as the control of disassembly damages are discussed. In addition to the evaluation of sensor suitability for use on assembled engines, future work and further challenges will be addressed.

### 5.1. Suitability of the Measuring System

In order to enable the standardization and objectification of the inspection process for turbine blades of aero engines, a borescopic sensor design was developed to address the packaging requirements on the aero engine. Using this sensor design, precise metric measurement data for the condition assessment of turbine blades can be recorded quickly and automatically. Research in the laboratory demonstrated that a borescopic sensor is suitable for inspection through maintenance openings and can be applied for the measurement of turbine blades. Based on the researched prototypes, the basis for real engine inspection is laid. To automate the inspection process, however, the kinematically restricted measuring system still has to be inserted linearly into the engine and positioned precisely for measurement. This requires further mechanical fixings and alignment fixtures, which poses a further challenge due to the limited positioning capability on the assembled engine. This poses the risk that a measurement system cannot be used for all engines or engine stages. In addition, the control of the engine shaft must be synchronized with the measurement system, and the variable environmental conditions of the workshop must be taken into account. Moreover, the limited positioning capability of the sensor prevents the full measurement of the leading and trailing edges of the turbine blades. Areas on the rear side and the tip of a turbine blade are also outside the field of view. However, since most damages occur at the leading and trailing edges (within the field of view), these can be measured and assessed.

Within the scope of sensor evaluation for in situ use, previous evaluations of borescopic sensor characteristics have shown that the separation of measuring head and camera-board and projector allows a good positioning of the measuring system and function robustly [18]. This enables precise optical measurements in areas that are difficult to access or were previously inaccessible. However, this Chip-on-the-Tip sensor design also implies many challenges [22]. The combination of a comparatively large working distance and the associated large measurement volume result in weak and uneven illuminated measurement scenes when using endoscopic 3D measurement systems. In addition to the increased noise of the miniaturized cameras, there are also no high-quality optics available such that inexpensive injection-molded lenses are applied. Moreover, these are not designed for these small working distances, so that the lenses are operated outside their designed depth of field range. In combination with the strong distortions of miniaturized lenses and borescopes, this leads to a limited reconstruction quality. Furthermore, the gravity-based bending of the borescope must be taken into account during the rotation of the borescopic measurement system. In experiments, it was shown that the rotation angle of the borescope has an impact on the reconstructed points. Therefore, the rotation of each measuring pose has to be calibrated or a general black box calibration has to be performed [18].

Nevertheless, with the continuous advancement in the field of smartphone cameras and the associated lenses, there is a great potential for optimizing the currently limiting camera sensor.

### 5.2. Film-Cooling Hole Detection and Point Cloud Evaluation

The detection of film-cooling holes forms the basis for the point cloud registration approaches and subsequent deviation analysis and must therefore be robust. In the context of the available and investigated turbine blades, the functionality of the film-cooling hole detection approach could be demonstrated at different wear conditions. In particular, the RANSAC-based pose estimation approach enables a robust point cloud registration although some film-cooling holes are detected falsely. In a further step, the separation of individual turbine blades based on a clustering approach will be developed and applied a priori to the feature detection. Thus, only features of a single turbine blade will be considered for the pose estimation, which results in a lower outlier quantity. In Figure 8a, it can be seen that features on the adjacent blades were also detected but due to outlier filtering, up to 50% of the segmented features can be erroneous. In addition, 2D image based feature segmentation approaches could be implemented to identify film-cooling holes. By means of the camera–borescope pair, classical image processing approaches can also be applied.

For the estimation of the registration quality of the presented multi-level feature-point cloud registration approach, the resulting deviation map of Figure 12a is considered. Here, an endoscopic measurement using the presented pose estimation was registered onto a reference measurement of the same component, and point deviations were determined. It is evident that the area within the blade could be fitted accurately but deviations occur in the area of film-cooling holes, trailing edge and parts of the leading edge. On average, the point deviation from the endoscopic measurement to a reference measurement (by means of an industrial sensor) is 16 μm. High deviations (up to 0.5mm) in the area of film-cooling holes are not meaningful since the film cooling holes are artificially closed during data processing and model derivation within the industrial software. Additionally, these can be worn or clogged within the course of operation. Due to the arrangement of the turbine blades inside the engine relative to the measurement system, the trailing edge of the turbine blade is about 2 cm further into the measurement volume than the leading edge, which in combination with the poor illumination results in higher noise and increased measurement uncertainty. This and the low feature count detected on the trailing edge, are a potential cause for misalignment. In addition, the achievable registration quality depends on the quality of the measurement data. Since the endoscopic point clouds are very noisy, this can be limiting for the registration process and may be a reason for the high deviations at the trailing edge. Nevertheless, it could be shown that the registration approach works across different scales, sensors and reference geometries. Applied to the small measurement volume of the endoscopic sensor as well as to the large-scale measurement of the industrial sensors, the registration approach works on the sensor independently and robustly.Thus, the applicability of this approach is possible beyond its use for pose estimation of turbine blades. Based on the presented registration approach, the determined geometric deviations can vary slightly. This may cause minor potential damage, such as cracks and coating damage, to avoid being properly evaluated. Furthermore, there are no common threshold values for the evaluation of the individual damages, so a metric evaluation was not pursued further. Damage that is expressed by material deformation/missing material, such as necks, dents and burnings, can, however, be identified reliably.

### 5.3. Evaluation of Component Protection

In order to investigate the component protection, load limits were determined experimentally. It was intended to show how much force can be applied to the blade root during disassembly without causing damage. As shown in the test and measurement series, no plastic deformation could be detected up to a force of 50 kN using laser confocal scanning microscope examination. During visual inspection, traces of the exerted load were apparent at a force of 40 kN. However, the deformation resistance and mechanical properties can also vary according to the age and state of wear of each blade. An exact assessment and acceptance of the force limits can only be made in agreement with and under the manufacturer’s supervision.

In this context, the possibility of fast inspection of each individual part was also investigated. Comparable to a visual acceptance and reject test, a (borescopic) fringe projection system can be used to obtain repeatable measurements of the blade root. By means of a standardized procedure, a plane fit of the undamaged surface of the blade root can be applied as well as the calculation of the respective point to plane distances. Thus, a simple and therefore robust deviation threshold comparison can be established. The implementation of this verification method is also possible with commercial sensors and software from, for example, Carl Zeiss GOM Metrology GmbH (Braunschweig, Deutschland).

## 6. Conclusions

In this paper, a borescopic 3D sensor based on the fringe projection profilometry was presented. With this sensor, the application and reliability of the optical inspection approach on aircraft engine components, such as turbine blades, were investigated before aiming for further industrialization of these sensors. For this purpose, the geometric characterization of difficult-to-access turbine blades through maintenance openings was investigated, as well as the inspection of disassembly damages at blade roots.

Based on a novel feature detection approach for film-cooling holes, a pose estimation algorithm for turbine blades assembled within an aero engine was demonstrated. By applying the presented pose estimation and registration methods to fringe projection measurements of different scales and reference geometries, it could be shown that this approach is universally applicable. In addition, a precise geometric deviation analysis was performed, with which damage and its location on the turbine blade can be determined metrically and objectively. Using the presented sensor and registration approach, geometry measurements of turbine blades with an average point deviation of 16 μm could be obtained with respect to industrial reference measurements in disassembled condition. In the case of defective turbine blades, the engine is disassembled, and all blades have to be removed from the turbine disk. This can result in non-permissible plastic deformations at the blade root, which is why each blade root must be inspected after disassembly, and an accept or reject examination must be carried out. By means of the borescopic sensor, a quick inspection of the blade roots could be implemented. In addition, exemplary force limits for component-friendly disassembly were determined using a laser scanning confocal microscope.

## Figures and Tables

**Table 1:** Components and its device numbers of the borescopic sensor.

Component	Device Number	Manufacturer
Camera sensor	OV2740	OmniVision Technologies, Inc. (Santa Clara, CA, USA)
Camera module	MP-FPC31105-18350-200	MISUMI Electronics Corp. (New Taipei, Taiwan)
Frame grabber board	See3CAM_CX3RDK	e-con Systems India Pvt Ltd. (Chennai, Tamil Nadu, India)
Borescope	86290CF	KARL STORZ SE & Co. KG (Tuttlingen, Germany)
Borescope lens	20200043 C-MOUNT lens	KARL STORZ SE & Co. KG (Tuttlingen, Germany)
Projector	DLP 4500 EVM	Texas Instruments Inc. (Dallas, TX, USA)

**Table 2:** Set force values for the experimental investigation.

Label	P51	P10	P40	2	P48	N26	-	-
**Force in kN**	20	30	40	50	60	70	80	100
**Surface Pressure in MPa**	44.2	66.3	88.4	110.0	132.6	154.7	176.8	221.0

## Data Availability

For reasons of confidentiality, the measurement data of the borescopic sensor cannot be published by agreement with the industrial cooperation partner.

## Abbreviations

    The following abbreviations are used in this manuscript:

CAD	Computer-Aided Design
DB Scan	Density-Based clustering procedure
DMD	Digital Micromirror Device
HDR	High Dynamic Range
ICP	Iterative Closest Point
K-NN	K-Nearest Neighbor
MIPI	Mobile Industry Processor Interface
RANSAC	Random Sample Consensus
SX	Single Crystal
